# Auditory Brainstem Implants: Recent Progress and Future Perspectives

**DOI:** 10.3389/fnins.2019.00010

**Published:** 2019-01-29

**Authors:** Kevin Wong, Elliott D. Kozin, Vivek V. Kanumuri, Nicolas Vachicouras, Jonathan Miller, Stéphanie Lacour, M. Christian Brown, Daniel J. Lee

**Affiliations:** ^1^Department of Otolaryngology, Icahn School of Medicine at Mount Sinai, New York, NY, United States; ^2^Department of Otolaryngology, Massachusetts Eye and Ear, Boston, MA, United States; ^3^Department of Otology and Laryngology, Harvard Medical School, Boston, MA, United States; ^4^Center for Neuroprosthetics, École Polytechnique Fédérale de Lausanne, Geneva, Switzerland; ^5^Department of Neurological Surgery, University Hospitals Cleveland Medical Center, Case Western Reserve University School of Medicine, Cleveland, OH, United States

**Keywords:** auditory brainstem implant (ABI), history, cochlear implant, neurofibromatosis type 2 (NF2), cochlear nucleus, cochlear aplasia, cochlear nerve hypoplasia, conformable electrode array

## Abstract

The auditory brainstem implant (ABI) was first developed nearly 40 years ago and provides auditory rehabilitation to patients who are deaf and ineligible for cochlear implant surgery due to abnormalities of the cochlea and cochlear nerve. The aims of the following review are to describe the history of the ABI and innovations leading up to the modern ABI system, as well as highlight areas of future development in implant design.

## Introduction

The auditory brainstem implant (ABI) is a neuroprosthetic device that provides hearing sensations to deaf patients who are ineligible for the cochlear implant (CI) due to anatomic constraints. The ABI bypasses the cochlear nerve to electrically stimulate second order neurons in the cochlear nucleus (CN) using a multichannel surface array in patients with cochlear and retrocochlear pathologies.

**FIGURE 1:**
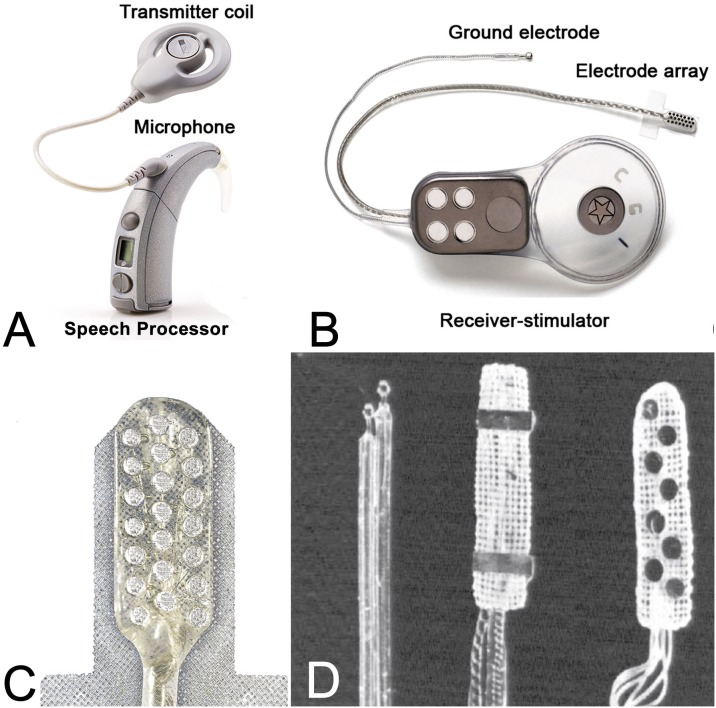
Components of the ABI. **(A)** External system consisting of a battery, microphone, speech processor, and transmitter coil and magnet, identical to the modern cochlear implant (CI). **(B)** Internal system includes a receiver-stimulator (that is affixed against the skull and above the craniotomy defect), a multichannel electrode array (that is placed through the lateral recess of the fourth ventricle), and a ground electrode (that is inserted against the calvarium and under the temporalis muscle). **(C)** Close-up of modern 21-electrode array. **(D)** Side-by-side comparison of three generations of ABI electrode designs. From left to right: *left*, platinum ball-style electrode implanted in the first patient in 1979; *middle*, two-plate platinum electrode with Dacron backing created in 1980; *right*, early multichannel ABI model with eight electrodes first used in 1992; constructed from platinum and medical grade silicone. Adapted with permissions from Brackmann et al. (1993).

The first ABI was developed at the House Ear Institute (HEI) in the 1970s (Edgerton et al., 1982). Similar to a CI, an ABI consists of an external ear-level worn device and an internal receiver-stimulator implant. The external system consists of a battery source, microphone, speech processor, transmitter coil, and magnet worn above and behind the ear (Figure 1A); the internal system consists of a surgically placed receiver-stimulator and magnet (in patients who require frequent magnetic resonance imaging (MRI), the magnet is removed and replaced with a metallic non-magnetic spacer at the time of ABI placement), a ground electrode, and an electrode array (Figure 1B,C). Unlike thin and flexible CI electrodes that follow the curved spatial arrangement of frequency selectivity in the cochlea (tonotopic axis), ABI electrodes are embedded in flat and rigid Silastic paddle that lies along the highly curved surface of the CN in the brainstem (Guex et al., 2015). Advancements have been made in the external speech processor and receiver stimulator technology but are otherwise identical to systems used for CIs. The basic design of the ABI multichannel array, however, has not changed in several decades.

Over one thousand ABIs have been placed in deaf adults and children around the world and is the most commonly placed surface stimulator in the central nervous system. The ABI was approved by the United States Food and Drug Administration (FDA) in 2000 for patients with Neurofibromatosis type 2 (NF2) and there are no audiologic criteria. Approximately one in every 25,000 individuals is diagnosed with NF2, and all NF2 patients will eventually experience profound hearing loss secondary to the tumor growth, radiation effects, and/or surgical resection of bilateral vestibular schwannomas (VS). This represents the largest clinical population in the United States that could potentially benefit from an ABI. Beyond NF2, expanded indications for ABI implantation have been explored in non-tumor adults and children and there are, a handful of clinical studies in the United States and abroad. Together these indications include bilateral cochlea and cochlear nerve aplasia and hypoplasia, traumatic nerve avulsion, and cochlear ossification accounting for an additional 2.1% of all individuals with profound sensorineural hearing loss who meet indications for an ABI (Kaplan et al., 2015).

The aims of the following review article are to (1) describe the history of the ABI and innovations leading up to the modern ABI system, and (2) review clinical outcomes with the ABI and highlight areas of future development in implant design and performance.

## Electrically Evoked Auditory Sensations and the Cochlear Implant

Concepts in electrically evoked hearing date back to the early 1800s, when Alessandro Volta, inventor of the modern battery, applied electrical current through metal probes to his ear canals, evoking sounds described as “crackling” or “bubbling” (Volta, 1982). A few decades later, French neurologist Guillaume Duchenne tested electrically evoked auditory sensations by using an alternating electrical current to stimulate the cochlea that was associated with a “buzzing” and “ringing” sound (Duchenne and Poore, 1883).

One of the earliest studies to elicit auditory sensations was performed in 1957, when Djourno and Eyries restored hearing sensations in a deaf patient by directly stimulating the cochlear nerve (Djuorno and Eyries, 1957). Electrodes were placed on the cochlear nerve and sound sensations were successfully generated when a current was passed through the electrodes. The patient was able to discriminate sound intensity and recognize limited closed-set speech. Inspired by this early work, Dr. William F. House and Dr. John Doyle implanted deaf patients with an early CI prototype that consisted of a single ball electrode. Outcomes from these initial CIs were encouraging, with patients obtaining both frequency discrimination and closed set speech recognition. Together, these early observations underscored the feasibility of electrically evoked auditory percepts and laid the foundation for the development of early ABI devices.

## Initial Applications in Neurofibromatosis Type 2

Neurofibromatosis type 2 or NF2 is a devastating autosomal dominant genetic syndrome characterized by mutations of the *NF2* tumor suppressor gene leading to an increased risk for central nervous system tumors including bilateral VS, meningiomas and ependymomas (Evans, 2009). VS are benign tumors that arise from the vestibular component of the vestibulocochlear nerve. VS are associated with hearing loss through these mechanisms: (1) tumor growth in the internal auditory canal and cerebellopontine angle, (2) iatrogenic injury during tumor resection (Sughrue et al., 2011) or (3) following radiation therapy.

Patients with NF2 often develop postlingual profound hearing loss as their disease progresses. Tinnitus, disequilibrium, and lower cranial nerve involvement (dysarthria, dysphagia, and hoarseness) are also common (Matthies and Samii, 1997). Due to retrocochlear hearing loss, deaf NF2 patients with cochlear nerve damage are not candidates for traditional CIs except in unusual circumstances (Peng et al., 2018). Thus, since the ABI bypasses the auditory periphery and stimulates the CN, NF2 patients were identified as an ideal population that could benefit from this device.

In 1979, Drs. William House and William Hitselberger performed the first ABI implantation on a 51-year-old woman with NF2 (Hitselberger et al., 1984). During surgery to remove the VS, a depth electrode was concurrently placed into the substance of the CN. This early ABI model was designed with a ball-style electrode connected to an external Bosch hearing aid processor (Figure 1D). These bipolar electrodes were connected to a single channel system, and together provided electrical stimulation across the CN. The patient recovered from surgery and received meaningful auditory sensations, including environmental sound awareness and improved lipreading (Eisenberg et al., 1987).

In 1980, researchers at the Huntington Medical Research Institute (Pasadena, CA, United States) developed an updated ABI device using a House/3M CI processor and two-plate electrode design made from polyethylene terephthalate mesh, which improved the handling and positional stable in animal models (Bullara et al., 1979; Figure 1D). Two years after initial implantation, the first patient underwent revision surgery due to lower extremity side effects and loss of auditory sensation attributed to a shift in the ball-style electrodes from their original position. For the revision, the patient was implanted with a new ABI device. The patient recovered well from revision surgery, benefited from the ABI, and no longer experienced any further shift in array position (Eisenberg et al., 1987). This patient continues daily use of the ABI device three decades later (Colletti et al., 2012).

### Single-Channel ABI System

From 1979 to 1991, 25 NF2 patients at HEI were implanted with a single-channel ABI system with dual-ball electrodes in the first patient, a two-plate electrode design in the next 19 patients, and a three-plate electrode design in the last five patients (Brackmann et al., 1993). Among this initial ABI cohort, three patients required revision surgery for device failure. Some users also experienced various minor technical and medical difficulties. Two patients experienced broken wires; four patients experienced percutaneous electrical plug connector infections; two patients developed postoperative meningitis treated with antibiotics; nine patients developed cerebrospinal fluid leaks treated with mastoid pressure dressings, surgical exploration and repacking, or lumbar drainage (Brackmann et al., 1993). Audiometric outcomes were promising, with the majority of patients receiving environmental sound awareness and obtaining subjective auditory benefit from their ABIs.

### Multi-Channel ABI System

These early patient experiences, along with parallel developments in CIs, prompted the development of a multichannel ABI by Laszig et al. (1991) in collaboration with Cochlear Ltd. (Sydney, Australia). With a multichannel system, different frequencies could be coded based on changes in the *pattern* of electrode activation, increasing programming flexibility. Among other design updates, the new device also included a mesh-style electrode array with three platinum plates mounted on polyethylene terephthalate mesh. Two versions of this device were produced (Laszig et al., 1999): a European model with 21 electrodes (Nucleus 22) and a North American model with eight electrodes (Otto et al., 2002; Figure 1D).

Starting in 1992, the Nucleus multichannel ABI device (Cochlear Corp., Englewood, CO, United States) replaced older ABI models in North America and abroad. Between September 1992 and October 1996, 27 NF2 patients with bilateral VS underwent ABI surgeries at the University of Verona, in Verona, Italy representing the first cohort to receive the new multichannel ABI device (Nevison et al., 2002). There were no major surgical complications and audiometric outcomes were better than single channel systems, with most ABI users achieving environmental sound awareness and improved pattern recognition for lipreading; two users were capable of understanding everyday conversation without lipreading, an achievement not seen with the earlier single channel systems.

In North America, multichannel ABIs were first implanted by surgeons at HEI (Schwartz et al., 2003). Starting in 1992, a total of 71 NF2 patients were implanted with the 8-electrode (North American) multichannel ABI. There were no major surgical complications; two patients developed CSF leaks treated with pressure dressings and lumbar drainage. Consistent with European outcomes, most ABI users achieved environmental sound awareness and the majority scored above chance on closed-set word recognition. Together, outcomes in the United States and in Europe demonstrated the safety and efficacy of multichannel ABI systems. These studies paved the way for FDA approval in 2000 of the Nucleus 24 ABI and ABI 541 (2016) for use in NF2 individuals ages 12 and older (Otto et al., 2002). The launch of the newest ABI (ABI541) was announced in February 2016, which improved upon older models with a newer receiver/stimulator and removable magnet, supporting MRI usage.

Recent outcomes have shown that the majority of NF2 patients implanted with the ABI achieve some phoneme discrimination and environmental sound awareness, however, only a minority obtain open set speech discrimination (Ramsden et al., 2016). Indeed, ABI outcomes overall remain inferior compared to the traditional CI. Only a few ABI users achieve open-set speech, and the majority rely on lip-reading and sign language for their communication needs. Most ABI users report only environmental sound awareness. Brainstem trauma has been identified as a major factor in the variability of ABI outcomes in NF2 patients, and surgical position, length of deafness, number of pitch electrodes, perceptual levels, and ABI stimulation rate all correlate to speech recognition in these patients.

An early improvement to ABI surgery was the use of intraoperative monitoring to guide placement of the ABI electrode array (Waring, 1995). Monitoring is essential because the array is placed in a “blind” fashion without direct visual cues to the position of the CN. Intraoperative monitoring relies on far field electrically evoked auditory brainstem responses (EABRs) that are obtained by delivering single, biphasic pulses to the ABI array through a processor and coil, and responses are recorded using a vertex (+) to nape (–) schema with the ground electrode at the hairline. Responses to stimulation of electrode pairs in the electrode array help determine the ideal paddle position most likely to elicit auditory precepts from the CN. Intraoperative EABRs are used to adjust array position and ensure the array has not shifted during surgical closure of the dura and soft tissue (Puram et al., 2015). However, even with EABR monitoring, the resulting positions of the electrode arrays are highly variable from patient to patient (Barber et al., 2017). This was shown by three-dimensional multiplanar reconstruction of adult and pediatric subjects. This novel study that classified the precise position of ABI electrode array in children and adult users demonstrated that this position varied widely among ABI users, with some orientations associated with improved perception and others associated with increased side effects (Barber et al., 2017). These observations lay the foundation for future efforts to use intraoperative navigation to help improve ABI placement.

### Penetrating Electrode Auditory Brainstem Implant

Another strategy to improve the ABI was the development of the penetrating electrode ABI, or PABI. The PABI is a hybrid consisting of microelectrodes and standard surface electrodes. Microelectrodes are designed with varying lengths to take advantage of the tonotopic organization of the CN and penetrate deep (1–2 mm) to stimulate neural pathways inaccessible at the surface. Development began in the early 1990s at HEI in collaboration with Huntington Medical Research Institutes (Pasadena, CA, United States) and Cochlear Ltd. (Sydney, Australia) and the final product consisted of 8 or 10 penetrating microelectrodes with 10 or 12 surface electrodes, respectively. The PABI was solely used as an experimental device in a clinical trial. In the trial of 10 NF2 patients, the PABI provided lower thresholds and greater selectivity of electrical stimulation (Shannon, 2012). However, clinical outcomes were variable and there was no significant difference in speech understanding between patients with PABIs and multichannel ABIs. Additionally, less than 25% of penetrating electrodes resulted in auditory sensations compared to 60% of surface electrodes (Otto et al., 2008). The PABI was discontinued because of serious side effects.

### Audiological Considerations

ABIs are programmed using a combination of monopolar and bipolar stimulation (Herrmann et al., 2015). With monopolar stimulation, psychophysical thresholds for each individual electrode in the array are measured in an ascending method starting at current levels below perception. Once auditory sensations are evoked, loudness comfort limits are determined by raising the current level from threshold to the level at which the subject first experiences uncomfortable loudness or side effects such as tactile stimulation, dysgeusia, or dizziness. For bipolar stimulation, electrical stimuli are delivered using biphasic single pulses that alternate in polarity. The phase of biphasic pulses alternates temporally between anodic and cathodic (13 pps, 150 μs phase duration, 8 μs interphase gap) to provide optimal cancellation of stimulus artifact during electrophysiologic testing. Specific bipolar electrode pairs are selected with a goal of sampling a variety of areas on the electrode pad. Similar to monopolar stimulation, psychophysical thresholds are measured for each bipolar electrode pair using an ascending, bracketing method starting at current levels below perception. The loudness comfort limits are measured for each electrode pair by raising the current level from threshold until the subject first reports uncomfortable loudness or side effects. Results from monopolar and bipolar perceptual testing are used to map electrodes and electrode pairs based on sensations elicited, and are integral to improving hearing outcomes with the ABI. Programming techniques vary across ABI centers due to local differences in training/expertise, equipment availability, and patient demographics (ex: children vs. adults).

### Cochlear Implantation in NF2 Patients

ABIs represent a major success in restoring a sense of hearing to NF2 patients with bilateral deafness. However, because ABI outcomes remain inferior compared to the traditional CI, in recent years, cochlear implantation has also been revisited as a treatment option in patients who have preserved continuity of the cochlear nerve. Kveton et al. (1989) reported the first staged CI in a patient who previously underwent VS resection using the translabyrinthine approach. Postimplantation performance was comparable to performance in non-tumor post-lingual implant recipients, which underscored the viability of auditory stimulation in this patient population. A few years later in 1995, the first simultaneous cochlear implantation with VS removal was performed by Arriaga et al. with results supported by similar early reports (Arriaga and Marks, 1995; Ahsan et al., 2003). Normal function of the CI and improved hearing postoperatively in these patients suggested that enough cochlear nerve fibers were preserved to allow for adequate stimulation by a CI, despite tumor pressure and surgical dissection. Since these case reports, several series have corroborated favorable audiometric outcomes in select NF2 patients who receive CIs (Lloyd et al., 2014), with some studies demonstrating up to 70–85% of patients achieving open-set speech discrimination (Carlson et al., 2012; Lassaletta et al., 2016). In a literature review of 108 patients with cochlear nerve deficiency, 25% achieved open set speech perception, 34% attained closed-set speech perception, and 41% achieved sound detection, suggesting that CI instead of ABI may be a beneficial in select NF2, with the potential of lower morbidity (Vesseur et al., 2018).

Successful hearing rehabilitation in NF2 patients using CI depends on careful preoperative assessment to confirm a viable cochlear nerve. Several tests exist to evaluate the functionality of the cochlear nerve. These include evoked compound action potentials (eCAPs) to determine the responsiveness of the cochlear nerve to cochlear stimulation and cochlear nerve action potentials (CNAPs), which may have greater predictive power when combined with fast ABRs.(Piccirillo et al., 2008). Intraoperatively, ABRs and EABRs can also be used for cochlear nerve monitoring as previously described. Postoperatively, promontory stimulation can be used to test the integrity of the cochlear nerve after VS resection. A review of these various tools to test the functionality of the cochlear nerve are reviewed by Lassaletta et al. (2017).

## Outcomes of the ABI in Non-Tumor Patients

Colletti et al. (2005) postulated that patients who were deaf from non-tumor cochlear or cochlear nerve pathologies could also benefit from ABIs. Both congenital and acquired conditions can disrupt the normal structure or function of the cochlea and cochlear nerve. Examples include congenital cochlear nerve aplasia, temporal bone fractures that involve the cochlea or internal auditory canal, labyrinthitis ossificans from bacterial meningitis, and extensive cochlear otosclerosis (Sennaroglu et al., 2011; Merkus et al., 2014).

Interestingly, early clinical outcomes data in these patients suggest that non-tumor ABI users may benefit more than NF2 patients. For example, while only a small minority of NF2 patients are capable of open-set sentence recognition, a significant number of non-tumor patients achieve open set speech perception (Colletti and Shannon, 2005). In four patients with cochlear ossification, three achieved open-set sentence recognition and could engage in normal conversation, and in six patients with head trauma (four with bilateral labyrinthine fractures and two with bilateral temporal bone fractures) all achieved closed-set word recognition, with three achieving open-set speech recognition and two even able to engage in everyday oral conversation (Colletti et al., 2005).

### ABI in Children

Kaplan et al. (2015) estimated that 2.1% of all deaf children in the United States have bilateral cochlea or cochlear nerve aplasias, making them ideal candidates for ABI surgery. Providing early sound perception is important in children due to cortical plasticity and critical development of central auditory processing (Kaplan et al., 2016). The first three non-tumor individuals implanted with the ABI were prelingually deaf children born with bilateral cochlear malformations and cochlear nerve aplasia (Colletti et al., 2002). After surgery, all children achieved environmental sound awareness, one achieved speech recognition, and another achieved moderate speech detection. The first ABI patient with auditory neuropathy was also a child (Colletti et al., 2004). This patient was a 6-year-old with severe congenital sensorineural hearing loss. As early as 2 months after activation, the child demonstrated environmental sound awareness and word detection.

Over the past few years, indications have continued to expand. In a recent study of patients with inner ear malformations, Sennaroğlu et al. (2016) found that among 60 children (12–64 months) with severe inner ear malformations, 46.7% achieving closed set discrimination and 20% developing open set speech discrimination after receiving the ABI. In the United States, there are three active FDA trials (Massachusetts Eye and Ear, New York University, and Children’s Hospital Los Angeles) recruiting pediatric candidates for the ABI. In the most recent update of the clinical trial in Boston, both primary and revision ABI surgery in children were performed successfully without any major or minor complications (Puram et al., 2016). All children achieved environmental sound awareness, and several demonstrated babbling and mimicry. This was the first study in the United States to report outcomes following pediatric ABI surgery, and results were consistent with studies performed abroad (Sennaroglu et al., 2009; Colletti et al., 2010).

Today, over 100 non-tumor pediatric patients have been implanted with ABIs in centers across the world (Noij et al., 2015). Surgical and audiometric outcomes vary but most pediatric ABIs overall experience meaningful auditory sensation and improved communication abilities. In some cases, pediatric ABI users with no neurodevelopmental concerns can achieve open set speech understanding (Sennaroğlu et al., 2016). Nevertheless, pediatric ABI users generally have inferior audiometric outcomes compared to CI users (Puram et al., 2016).

## Future Directions

Despite advancements in ABI technology over the past few decades, several challenges remain. A key limitation of current ABIs is the highly variable and often unpredictable audiometric outcomes; indeed, while some patients achieve open-set speech perception, the majority experience limited environmental sound awareness and speech pattern recognition (Colletti et al., 2005, 2009; Noij et al., 2015). The reasons for the variability in outcomes are not known. One explanation in tumor patients is that the tumor, or surgery to remove it, has damaged CN neurons that are important in coding for speech (Colletti and Shannon, 2005). Also, as mentioned earlier, positions of the ABI array vary from patient to patient (Barber et al., 2017). Finally, there are limitations of electrical stimulation, which is susceptible to channel cross talk, activation of competing pathways, and non-auditory side effects. These shortcomings may be even more pronounced with brainstem implants due to the unpredictable neural pathways in the CN compared to the highly tonotopic spiral ganglion that is stimulated by the CI.

### Optogenetics

**FIGURE 2:**
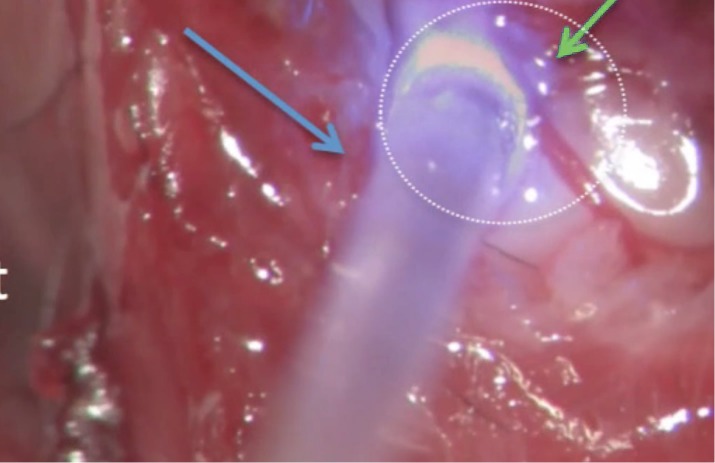
Surgical view of an optogenetic experiment that stimulates the photosensitized left dorsal CN (green arrow) using pulsed radiant energy in a mouse model. Optical stimulation is delivered by a flexible optical fiber (blue arrow) coupled to a blue light laser. Direct visualization of the dorsal CN was achieved using a posterior craniotomy as described by Kozin et al. (2015).

A novel strategy to improve brainstem implants is through the use of light to control genetically modified cells, a field known as *optogenetics* (Deisseroth et al., 2006). Compared to electricity, light has the advantage of increased specificity, which allows for selective stimulation and inhibition of neural pathways. To sensitize human tissue to visible light, a viral vector-mediated delivery of light-sensitive channel proteins is currently applied. One of the first studies to demonstrate the feasibility of optogenetically controlled auditory stimulation was performed by Darrow et al. (2015) who used adeno-associated viral gene transfers to express channelrhodopsin-2 (ChR2) in a murine model (Darrow et al., 2015; Kozin et al., 2015). Using an optical fiber coupled to a blue light laser directed at the infected dorsal CN, excitatory spiking activity was achieved in the inferior colliculus and auditory cortex (Figure 2).

In 2014, a new channelrhodopsin called *Chronos* was isolated by Klapoetke et al. (2014) from the algal species *Stigeoclonium helveticum*. Testing showed that *Chronos* had high light sensitivity and faster channel kinetics than any previous channelrhodopsin, making it ideal for optogenetically based auditory implants. Since its discovery, *Chronos* has continued to show promise in both *in vivo* and behavioral studies, and is capable of a wide range of temporal stimulation rates for better discriminability in auditory neural circuits (Shimano et al., 2013; Guo et al., 2015). In one experiment, light-evoked responses of the older ChR2 opsin were compared with *Chronos* in a murine ABI model (Hight et al., 2015). Results showed that *Chronos* activated the inferior colliculus at higher stimulation rates than ChR2. As further research develops, opsin technology may support the evolution of future optogenetic-based brainstem implants.

### Conformable Electrode Arrays

**FIGURE 3:**
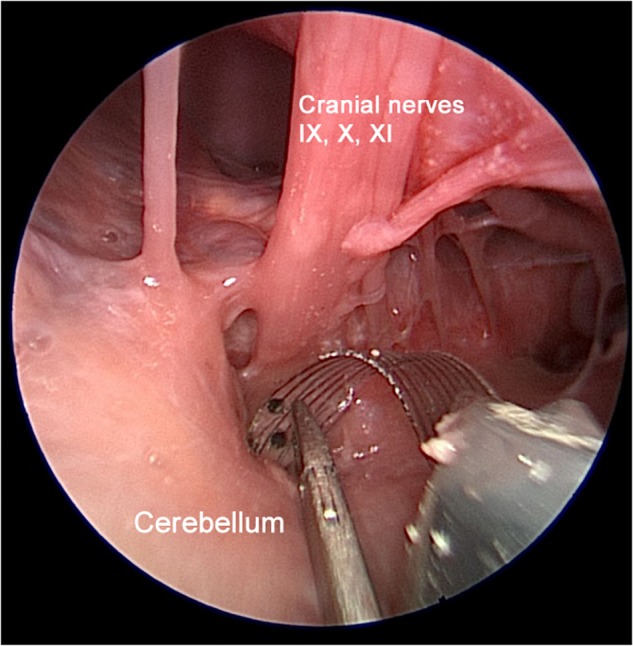
Multichannel flexible ABI arrays have been engineered and fabricated for testing in a collaboration between Massachusetts Eye and Ear and École Polytechnique Fédérale de Lausanne (EPFL). This photograph shows a conformable EPFL electrode array developed on thin polyimide substrate in an experimental rodent model (Guex et al., 2015). Unlike modern ABI technology, our designs will improve the electrode-tissue interface by conforming to the convexity of the CN. Adapted with permissions from Guex et al. (2015).

Modern electrode array designs are flat and result in suboptimal contact to the complex curvature of the CN and may explain the poor spectral resolution of most ABI users. Application of flexible polymers that conform to the brainstem surface has been an exciting area of research by our group (Minev et al., 2015; Figure 3). Among conducting polymers, poly(3,4-ethylenedioxythiophene) (PEDOT) has gained particular interest for its electrochemical stability, efficient charge transfer, and biocompatibility (Ludwig et al., 2006; Cui and Zhou, 2007). In a recent study, the electrode sites of conformable ABI electrode arrays were coated with PEDOT and polystyrene sulfonate (PSS); the conformable array allowed for greater access to the tonotopic axis of the CN in mice and the conductive polymer (PEDOT:PSS) provided ideal electrical (impedance, charge injection capacity) and physical characteristics (size, thickness, bendability) for CN stimulation (Guex et al., 2015). Studies are soon underway to characterize acute and chronic responses using conformable arrays in rodent and primate models.

### Electrode Array Position

Regardless of the type of stimulation employed, we must improve and standardize the placement of the stimulating array. The promising approach of combined CT and MRI imaging, which has been used in the auditory midbrain implant (Lim et al., 2007), may lead to a better understanding of electrode positioning and more consistent and favorable hearing outcomes in future ABI users. The combination is needed because CT has excellent resolution of the electrode array and bony structures (Barber et al., 2017) whereas MRI resolves the neural structures of the brainstem. This direction is needed to show which positions have a greater likelihood of CN activation and the best speech comprehension.

## Conclusion

Over the past four decades, the ABI has gone through numerous iterations in device and electrode design. While the ABI was originally designed for NF2 patients, recent studies suggest a possible role of this technology to provide hearing in non-tumor children and adults. While the ABI can restore meaningful sound awareness in most patients, outcomes remain modest compared to the average CI user. Improvements in electrode positioning and development of ABI surgical navigation methods, the development of new-generation conformable surface arrays, and optogenetic modulation of the CN neurons are active areas of research that may improve performance.

## Author Contributions

All individuals listed as authors in this manuscript met International Committee of Medical Journal Editors guidelines for authorship. KW acquired and analyzed the data, and drafted the manuscript. EK and DL conceived and designed the study, analyzed and interpreted the data, and drafted the manuscript. NV, VK, and MB analyzed and interpreted the data. JM conceived and designed the study. SL conceived and designed the study, and analyzed and interpreted the data. All authors reviewed and approved the final manuscript and agree to be accountable for all aspects of the work in ensuring that questions related to the accuracy or integrity of any part of the work are appropriately investigated and resolved.

## Conflict of Interest Statement

The authors declare that the research was conducted in the absence of any commercial or financial relationships that could be construed as a potential conflict of interest.
